# Elongation rate and average length of amyloid fibrils in solution using isotope-labelled small-angle neutron scattering†

**DOI:** 10.1039/d1cb00001b

**Published:** 2021-04-14

**Authors:** Ben J. Eves, James J. Doutch, Ann E. Terry, Han Yin, Martine Moulin, Michael Haertlein, V. Trevor Forsyth, Patrick Flagmeier, Tuomas P. J. Knowles, David M. Dias, Gudrun Lotze, Annela M. Seddon, Adam M. Squires

**Affiliations:** Department of Chemistry, University of Bath Bath UK A.Squires@bath.ac.uk; ISIS Facility, Rutherford Appleton Laboratory, STFC, Chilton Didcot OX11 0QX UK; MAX IV Laboratory, Lund University P.O. Box 118 Lund 221 00 Sweden; Life Sciences Group, Institut Laue Langevin 38042 Grenoble Cedex 9 France; School of Chemistry & Physics Keele University Staffordshire ST5 5BG UK; Department of Chemistry, University of Cambridge Cambridge CB2 1EW UK; Centre for Misfolding Diseases, University of Cambridge Cambridge CB2 1EW UK; Department of Chemistry, Physical and Theoretical Chemistry Laboratory, University of Oxford South Parks Road Oxford OX1 3QZ UK; School of Physics, HH Wills Physics Laboratory, Tyndall Avenue, University of Bristol Bristol BS8 1TL UK

## Abstract

We demonstrate a solution method that allows both elongation rate and average fibril length of assembling amyloid fibrils to be estimated. The approach involves acquisition of real-time neutron scattering data during the initial stages of seeded growth, using contrast matched buffer to make the seeds effectively invisible to neutrons. As deuterated monomers add on to the seeds, the labelled growing ends give rise to scattering patterns that we model as cylinders whose increase in length with time gives an elongation rate. In addition, the absolute intensity of the signal can be used to determine the number of growing ends per unit volume, which in turn provides an estimate of seed length. The number of ends did not change significantly during elongation, demonstrating that any spontaneous or secondary nucleation was not significant compared with growth on the ends of pre-existing fibrils, and in addition providing a method of internal validation for the technique. Our experiments on initial growth of alpha synuclein fibrils using 1.2 mg ml^−1^ seeds in 2.5 mg ml^−1^ deuterated monomer at room temperature gave an elongation rate of 6.3 ± 0.5 Å min^−1^, and an average seed length estimate of 4.2 ± 1.3 μm.

## Abstract 2

We demonstrate a solution method that allows both elongation rate and average length of amyloid fibrils to be independently determined.

## Graphical abstract

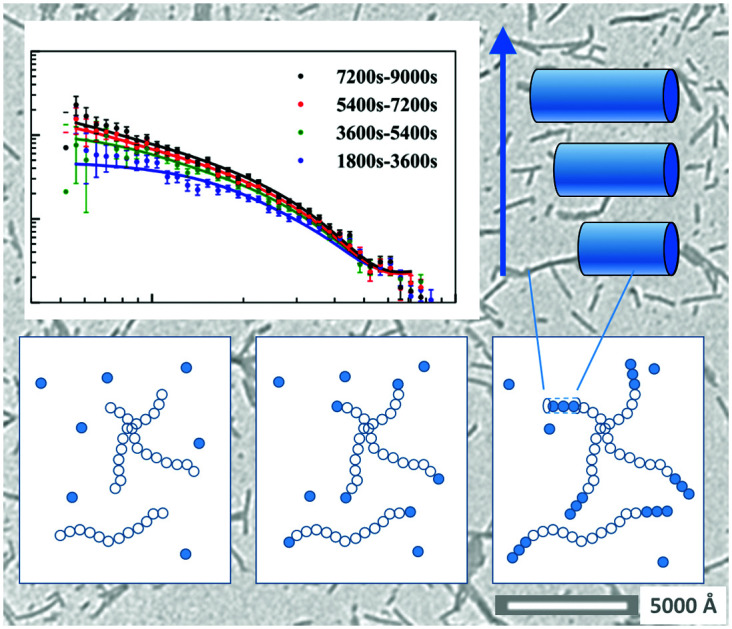

## Introduction

Amyloid fibrils are microscopic fibers that can self-assemble from a range of proteins and synthetic peptides. They have received considerable attention due to their links with diseases such as Alzheimer's and Parkinson's,^1^ but their study is also of interest to further our understanding of the fundamental thermodynamic and kinetic aspects of protein folding and self-assembly, and as potential nanomaterials.

Amyloid fibril formation is greatly accelerated by the addition of pre-formed “seeds”. This phenomenon is considered a model for prion disease propagation.^2^*In vitro* seeding experiments and their biological counterparts are of particular interest in the study of polymorphic fibrils that show “strain” behavior, where the same protein can form fibrils with different structures (“polymorphs”), and where the fibrils’ morphology in seeding experiments is seemingly “inherited” from the “parent” seeds. Inherited fibril morphology through seeded growth is thought to represent a mechanism for genetic inheritance in yeast prions, and the brittleness of the fibrils and their growth rate are both believed to play key roles in their physiological impact.^3^ In the case of fibrils grown from biological seeds extracted, for example, from brain samples, it has been shown that the fibril structure can differ from that of the seeds, suggesting that post-translational modification of α-synuclein and/or additional molecules are necessary for filament replication of seeds from disease *in vivo*.^4^

Currently, the most commonly used solution methods to probe the kinetics of amyloid fibril growth are based on circular dichroism or fluorescence spectroscopy, the latter based on dye-binding; light/small-angle scattering; and chromatographic approaches that monitor the decrease in the concentrations of the precursor monomeric protein as it is incorporated into growing fibrils.^5–9^ However, these techniques can only probe the overall percentage of protein in the fibrillised form *versus* the unfibrillised form, and the rate of change of these percentages. This rate of change is dependent on two different variables which the techniques outlined above cannot separate: the average rate of growth for a single amyloid fibril (“elongation rate”), and the number (and length distribution) of the amyloid fibrils. In the comparison of seeded growth experiments from two different amyloid protein types, the kinetics for one may be faster than the other, either because the elongation of individual fibrils is faster, or because there is a greater number of shorter fibrils present in the fibril seed stock solution; the techniques cannot distinguish between the two explanations.

Recent studies have aimed at the development of techniques that can overcome these problems by directly measuring the growth of individual amyloid fibrils. These studies have used techniques such as quartz crystal microbalance sensors,^10^ atomic force microscopy (AFM),^11^ and total internal reflection fluorescence microscopy.^12^ Single molecule techniques such as AFM have begun to emerge as methods that allow direct observation of the growth of amyloid fibrils and determination of their elongation rates *in situ*. However, these techniques also have their own limitations. A discrepancy between the morphology of fibrils formed on a mica surface during AFM experiments and fibrils formed in solution suggests that the elongation rate of the amyloid fibrils on a mica surface would not be representative of the fibril growth under solution conditions. It has been suggested that fibril growth on surfaces may be restricted. The surface properties of mica, such as hydrophobicity, hydrophilicity and charge, are known to affect the growth rate and morphology of the fibrils.^13–15^ Data can also be hard to analyze if the scan rate of the AFM experiment is close to or slower than the fibril growth rate. In addition, there are concerns that the AFM measurement may influence the kinetics through interactions with the probe tip.^16^

In order to determine the length of fibrils in the seed stock solution, techniques such as transmission electron microscopy (TEM) and AFM can be used to determine an average length, which can be combined with overall transformation measurements to determine elongation rates. However, these length measurements often tend to be quite subjective due to the selection of interpretable images for quantification.^17,18^ Furthermore, techniques such as TEM and AFM require the removal of an aliquot of sample and extensive sample preparation. Both techniques require deposition of a sample onto a surface, washing of the surface, and subsequent drying. If the measured length distribution is not representative of the bulk, then the results will be skewed; this can occur when some fibril lengths or polymorphs have a greater affinity to the surface than others.^19^ Also, the drying process can affect the morphology of the fibrils,^20^ and can disrupt fibrils into smaller fragments, thereby giving an inaccurate representation of the fibril lengths in the bulk solution.^12^

Here we present a new method to investigate elongation rates during amyloid fibril growth in solution, and also to determine the number (and hence average length) of the fibril seeds. The method exploits small-angle neutron scattering (SANS), used in conjunction with deuteration approaches. Advanced methods for deuteration of proteins and other macromolecules have been developed in the ILL's Life Sciences Group and are now widely applied in neutron applications including SANS, crystallography, reflectometry and dynamics.^21–29^ In the method we present here, neutron scattering patterns from deuterated growing ends are modelled as rods. The increasing lengths of the rods can then be determined over time and used to calculate an elongation rate for an individual fibril. In addition, the average length of the initial fibril seeds can be calculated, since the concentration of the rods is a measure of the concentration of fibril ends.

The information yielded from this SANS/deuteration methodology will be vital in improving the mechanistic understanding of amyloid and prion diseases, whilst also furthering the foundation of the theoretical understanding of peptide fibrillation kinetics based on molecular models.

## Results and discussion

### Seed characterization

**Fig. 1:**
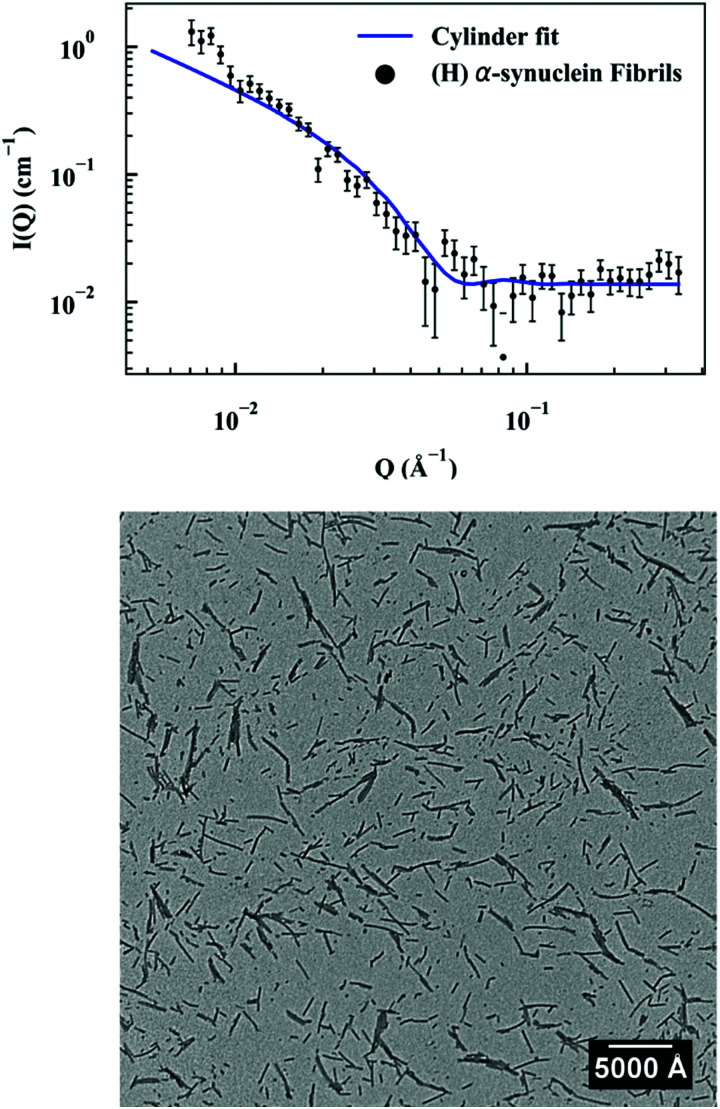
(top) Hydrogenated α-synuclein fibril seeds (1.2 mg ml^−1^) in 80% D_2_O (Sodium phosphate HPCE buffer pH 7.4, 10 mM) with cylinder fit, radius 60 Å. (bottom) TEM image of α-synuclein fibril seeds. Scale bar represents 5000 Å.

First, a model of the fibrils dimensions was created, for use in subsequent analysis, using the scattering arising from hydrogenated α-synuclein seeds in a contrasting deuterated solution. A cylinder model was used to fit the scattering curve from the 1.2 mg ml^−1^ hydrogenated α-synuclein fibril seeds in 80% D_2_O (Fig. 1).^30^ The model corresponds to a cylinder with radius constrained to be 60 Å (based on TEM measurements, ESI,† Fig. S1), and length that was allowed to vary in the fitting process. This analysis effectively returned infinite lengths, since a SANS experiment can typically only resolve length scales up to 1000 Å. A mean diameter of 120 ± 20 Å (*n* = 100) and a mean length of 2790 ± 1250 Å (*n* = 66) was calculated from TEM (Fig. S1, ESI†). α-Synuclein seed fibrils exhibited a ‘twisted’ morphology and other distinct fibril morphologies were not observed. The agreement between the experimental data and the fit is reasonable. At low *Q* there is a divergence of the experimental data from the model, which could be attributed to higher order aggregation such as fibril–fibril association.^31^

In addition to providing a model for subsequent analysis, the scattering curve from the seeds can be used to determine the mass-per-unit-length (MPUL) of the α-synuclein fibrils; a parameter that can differentiate between fibril morphologies and provides insight into the structure. The MPUL can be calculated by first determining the *y*-intercept *I*_c_(0) of a modified Guinier plot (ESI†), arising from hydrogenated α-synuclein fibril seed scattering (9.0 ± 0.5 × 10^−4^ cm^−1^ Å^−1^) and applying it to the following equation:^16^
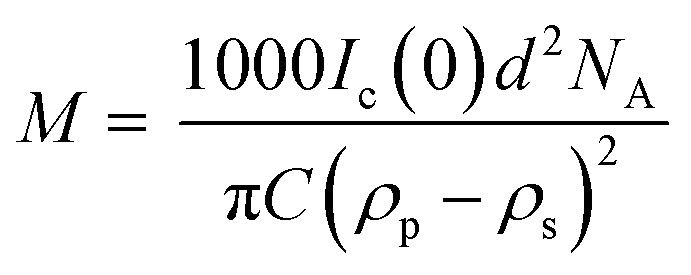
where *M* is the mass-per-unit-length of the fibril, *d* is the mean density of a protein (1.35 g ml^−1^), *N*_A_ is Avogadro's number (6.022 × 10^23^ mol^−1^), *C* is the concentration of protein in the fibril seeds (1.2 mg ml^−1^),^32^*ρ*_p_ is the scattering length density of the fibril seeds (2.77 × 10^10^ cm^−2^) and *ρ*_s_ is the scattering length density of the solvent (4.99 × 10^10^ cm^−2^). The fibril scattering length density was calculated from the atomic composition of α-synuclein using the ISIS Biomolecular Scattering Length Density Calculator program (http://psldc.isis.rl.ac.uk/Psldc/). Using these values, the mass-per-unit-length of the α-synuclein fibrils was determined to be 4800 ± 300 Da Å^−1^. Dearborn *et al.* used cryo-electron microscopy and scanning transmission electron microscopy (STEM) to investigate α-synuclein fibrils reporting a mean mass-per-unit-length of 5910 Da Å^−1^.^33^ There is reasonable agreement between the mass-per-unit-length determined by STEM and SANS.

### Elongation rates

**Fig. 2:**
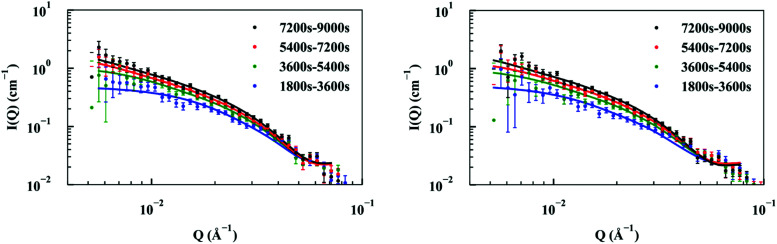
Repeat experiments showing kinetic SANS of hydrogenated α-synuclein fibril seeds at concentration 1.2 mg ml^−1^ after mixing with 2.5 mg ml^−1^ deuterated α-synuclein monomers (data points) in buffer contrast-matched to seeds; fits to patterns from growing deuterated ends at subsequent time points modelled as cylinders of radius 60 Å and variable lengths (solid lines).

Elongation experiments were performed in a solution containing 1.2 mg ml^−1^ hydrogenated seeds and 2.5 mg ml^−1^ deuterated monomer, in buffer containing 40% D_2_O, which contrast-matched the seeds (see ESI,† Fig. S3). As deuterated α-synuclein grew on the seed ends, the contrast with the solution gave rise to scattering signals (Fig. 2). The patterns were modelled as cylinders of radius 60 Å obtained as described in the previous section, and the length was allowed to vary as a fittable parameter. The best fits obtained over the range *Q* = [0.00562 to 0.07119 Å^−1^] for experiment 1 and *Q* = [0.0052 to 0.07688 Å^−1^] for experiment 2 are also included in Fig. 2.

**Fig. 3:**
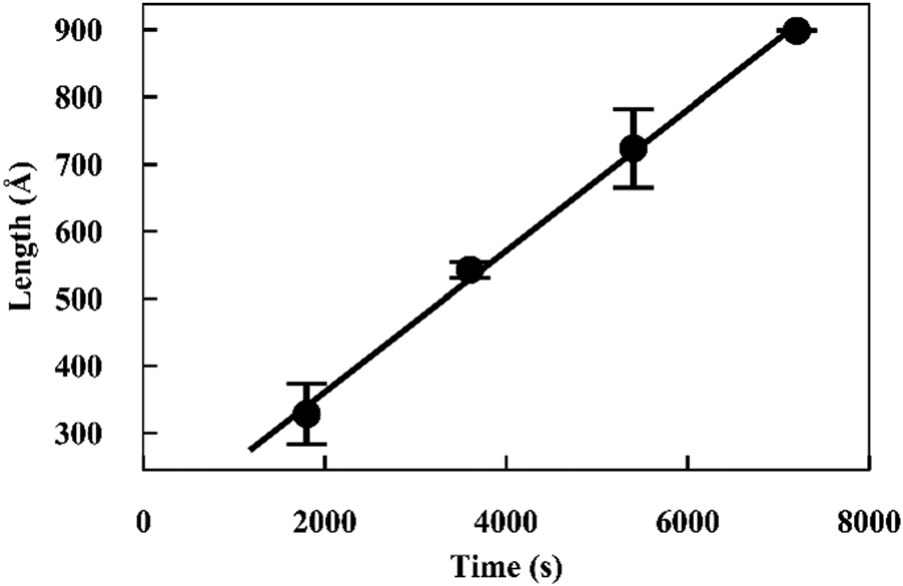
Plot of length *versus* time for α-synuclein fibril seed elongation. Length values were obtained from averaged cylindrical fits to data shown in Fig. 2.

The fits to the patterns from the deuterated α-synuclein ends give length values at subsequent time points, from which we obtain an averaged plot of length *versus* time (Fig. 3). A linear elongation rate can be extracted from the slope of the linear fit. The linear extension rate for α-synuclein fibrils was found to be 7.0 ± 0.8 Å min^−1^ in the first experiment and 5.6 ± 0.5 Å min^−1^ in the second experiment, giving an average of 6.3 ± 0.5 Å min^−1^ (20 mM phosphate buffer, pH 7.4, 25 °C, monomer concentration 2.5 mg ml^−1^). This result is in reasonable agreement with other reported elongation rates of amyloid fibrils. Buell and co-workers reported an average elongation rate of ∼10 Å min^−1^ for α-synuclein fibrils (20 mM phosphate buffer, pH 7.4, 37 °C, monomer concentration 0.29 mg ml^−1^) measured with fluorescence kinetics, with seed lengths determined by AFM, whilst Wördehoff and colleagues reported an elongation rate of 85 ± 37 Å min^−1^ for α-synuclein fibrils measured using total internal reflection fluorescence microscopy (20 mM 2-(*N*-morpholino) ethanesulfonic acid (MES) buffer, pH 6.0, 25 °C, monomer concentration 2.3 mg ml^−1^).^34,35^ Although our numerical value is similar to those of Buell *et al.*, the conditions are not exactly comparable: our results were obtained at an order of magnitude higher monomer concentration, but at 25 °C rather than 37 °C. Our results are an order of magnitude slower than Wördehoff's, but were obtained at pH 7.4 rather than 6.0, which Buell *et al.* suggest causes an order of magnitude decrease in elongation rate.^34^ The good agreement between elongation values from both of the kinetic experiments suggests the data from the SANS contrast matching technique is reproducible.

The good fit to a single cylinder length at each timepoint suggests that the growing ends are growing at the same rate. Fibril ends growing at different rates would show a worse fit to a single cylinder length, and would require fitting to the sum of two cylinder populations. For this situation we would be able to measure both elongation rates of the ends. Within the noise, we do not detect a difference in the rate of growth for each end.

### Concentrations of fibril ends

In addition to the linear extension rate, parameters such as concentrations of fibril ends and hence seed sizes can be extracted and validated using the kinetic SANS of α-synuclein fibrils. Using these values, it is possible to validate the elongation rate and monitor secondary kinetic processes.

The SANS coherent macroscopic scattering cross section for monodisperse particles in a solvent can be modelled as:^30,36,37^
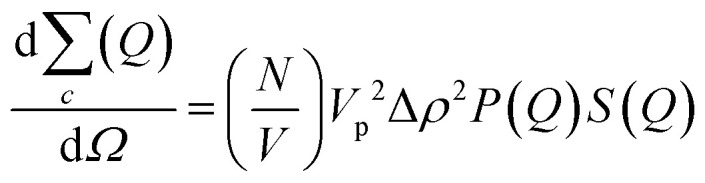
where 
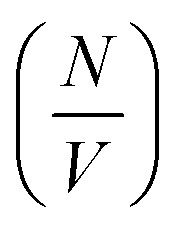
 is the number density of particles, *V*_p_ is the volume of the particle, Δ*ρ*^2^ is the contrast factor, *P*(*Q*) is the form factor and *S*(*Q*) is the structure factor.

For the case where *Q* = 0 and the solution is dilute, *P*(*Q*) = 1 and *S*(*Q*) = 1. The equation above simplifies to:^36,37^
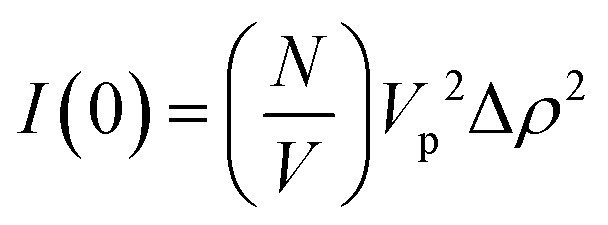
where *I*(0) is the scattering intensity at the zero angle.

As *I*(0) ∝ *V*_p_^2^, if the length of the seeds increases linearly with time then *I*(0) will have a squared relationship with time. The values of *I*(0) for each time point in the SANS kinetic experiment can be determined from a Guinier plot. Furthermore, using values for length of labelled ends obtained from the cylindrical curve fits (Fig. 2 and 3), a plot of length^2^*versus I*(0) will give a linear relationship.

**Fig. 4:**
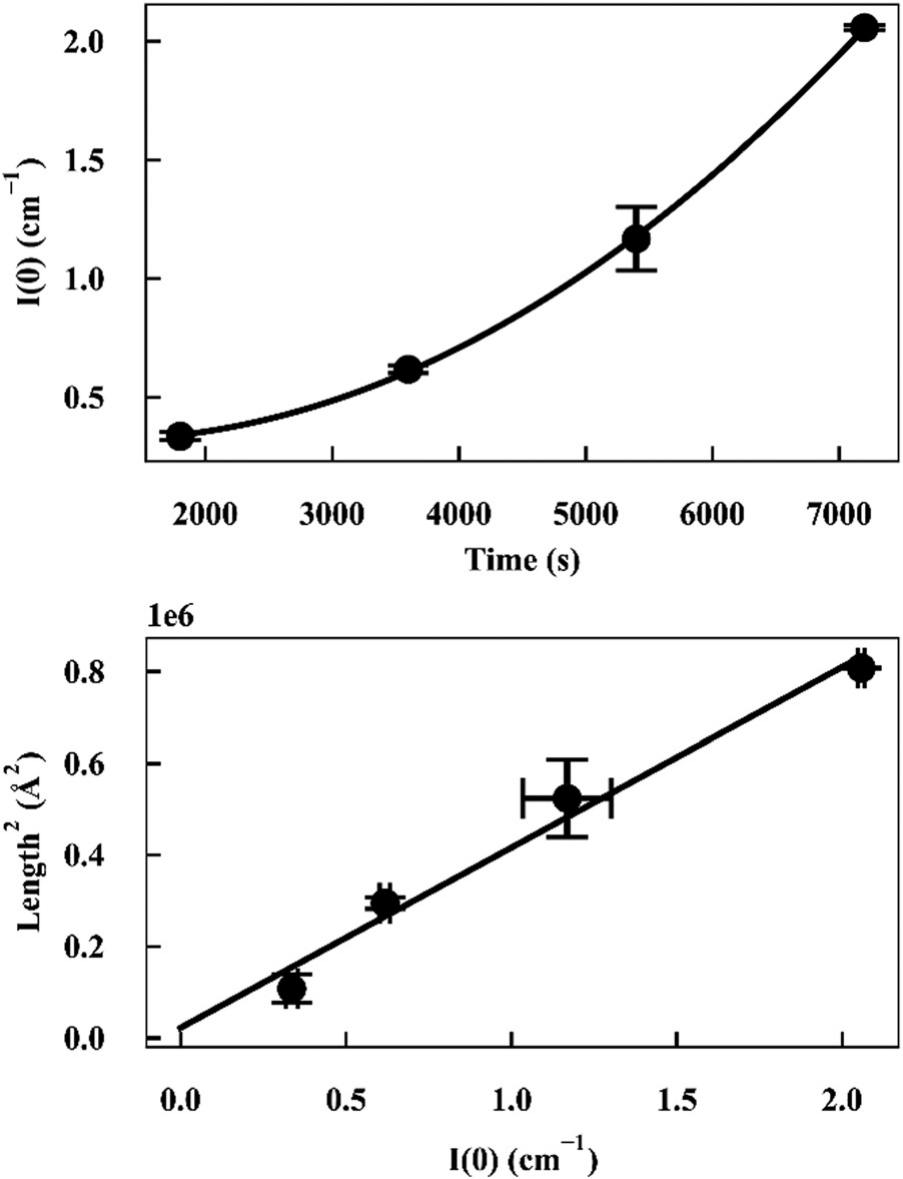
(top) Plot of *I*(0) *versus* time for α-synuclein fibril seed elongation. (bottom) Plot of length^2^*versus I*(0) for α-synuclein fibril seed elongation.

Deviation from these relationships would suggest a change in 
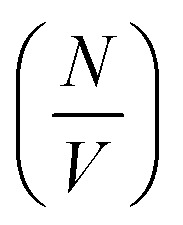
 or Δ*ρ*^2^. As there is not significant deviation from the expected relationships (Fig. 4), it can be inferred that there is no appreciable change of Δ*ρ*^2^ or 
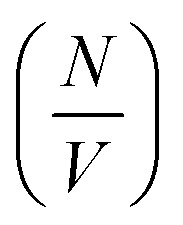
 over the time course of the experiment. The data are consistent with a constant value for 
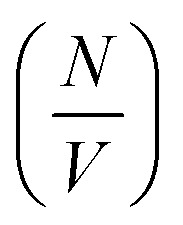
, the number of growing, labelled ends per unit volume. This would appear to argue against any significant breakage or secondary nucleation during the experiment within experimental error. The system can therefore be treated as a fixed number of growing ends.

Although the overall fibril seed length is too long to determine directly by the experiment, the average length can be estimated from the kinetic SANS data and the hydrogenated α-synuclein fibril seed scattering, using the fibril seed concentration, the mass-per-unit-length (MPUL) of the seed fibrils and the number density of seeds. The fibril seed concentration (in mg ml^−1^) is already known from the experimental preparation, and the MPUL of the seed fibrils was calculated previously using the non-matched hydrogenated α-synuclein fibril seed scattering (ESI†).

The values of 
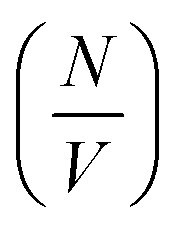
 determined previously gives an estimate for the number density of growing fibril ends:
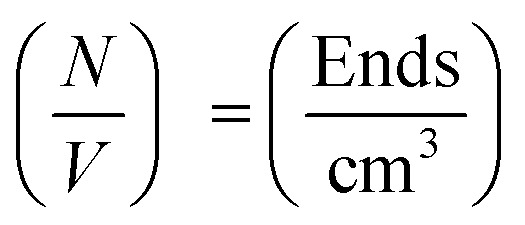


If the growth of the fibril ends is assumed to be bidirectional, as previously suggested, then there will be two growing ends for each seed.^38^ The number density of seeds can be determined as:
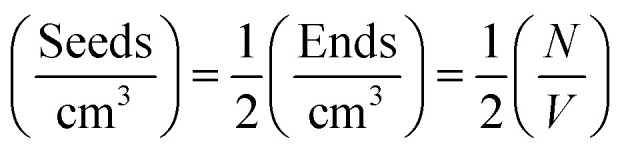


Using the number density of the seeds, the known concentration of seeds and the MPUL, the following equation gives an estimate for the fibril seed length:
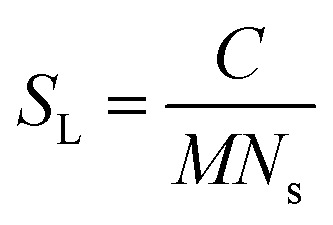
*S*_L_ is the fibril seed length (Å), *C* is the concentration of fibril seeds (mg ml^−1^), *M* is the mass-per-unit-length of the hydrogenated fibrils (g Å^−1^), and *N*_s_ is the number density of seeds.

The fibril seed lengths were estimated to be 3.9 ± 1.8 μm from the first kinetic run and 4.5 ± 1.9 μm from the second kinetic run, giving an average of 4.2 ± 1.3 μm. Fibril seed lengths determined by TEM (S1) gave a mean length of 280 ± 120 nm (*n* = 66). We speculate two possible reasons for this discrepancy: it is possible that not all of the fibril seed ends are active and accessible for growth (in our SANS experiment), which would lead to an underestimate of the number of ends, and therefore an overestimate of the average seed length; conversely, it is possible that the TEM is under-estimating the fibril seed lengths, either because sample preparation and dehydration for TEM experiments induces breakage, or because shorter fibril seeds have greater affinity for TEM grids than longer seeds, skewing the length estimate.

At later timepoints where the fibril ends are approaching a *Q* range where the length appears “infinite” *i.e.* is much greater than the interrogated *Q* space of the experiment, the error in determining the length of the growing ends, and *I*(0) increases inducing a large uncertainty in the estimation of the fibril seed lengths.

## Conclusions

This technique outlined here provided a new way of measuring amyloid fibril elongation rates. The average elongation rate of α-synuclein fibrils was determined to be 6.3 ± 0.5 Å min^−1^. The values reported by this procedure corresponded to results from previous research measured using fluorescence kinetics, where measurements were performed in solution (∼10 Å min^−1^) using an independent estimate of seed length obtained by TEM.^34^ Elongation measured on surfaces using total internal reflection fluorescence microscopy was faster (85 ± 37 Å min^−1^).^35^ Our technique does not require knowledge of seed lengths to determine elongation rate. Furthermore, as the technique utilizes solution scattering it does not require dye binding, nor does the sample have to be immobilized on a surface; these conditions are required for fluorescence kinetics and total internal reflection fluorescence microscopy and may influence the elongation rate.^13,34,35,39^

The technique is reasonably universal, and is currently limited by signal:noise, which will be overcome by increasingly powerful neutron sources. In the current experiment, these considerations prevent shorter acquisition times, which means that we would not be able to study fibrils with a greater elongation rate, such that sufficient scattering of the sample cannot be collected before the fibril lengths are too long to be accurately measured by SANS. The technique can in principle be applied to samples with structural polymorphism as the observed scattering patterns are a linear combination of scattering from each species. For example, if there were two polymorphs growing at different rates, it would be possible to fit the scattering patterns to two populations of cylinders and measure an elongation rate for each. This potential would represent an advantage over dye-binding or CD spectroscopic methods, where only a single value is obtained for both species. However, to realise this potential for SANS requires much greater signal:noise from a brighter neutron beam.

In addition, the data also provide an estimate of the number concentration of fibril ends and thus the average fibril length. The technique is applicable even for fibrils that are long, flexible and/or intrinsically curved, factors that present a challenge to other methods of estimating length.

With these two measurements, neutron scattering presents powerful tools to study dynamics of fibril formation, directly determining different contributions from breakage and elongation rates, and ultimately helping to shed light on amyloid processes involving disease and other prion strain propagation contexts.

## Experimental

### Protein production

α-Synuclein (hydrogenated and perdeuterated) was produced recombinantly in the PSB Deuteration Laboratory (D-LAB) platform within the Life Sciences Group at the ILL.^21^ A kanamycin resistant expression system coding for α-synuclein was transformed into BL21(DE3) cells. A high-cell density fed-batch culture using d_8_-glycerol (Euriso-top) as a carbon source was then carried out using a computer-controlled protocol at a temperature of 30 °C, a *p*D of 6.9, and a *p*O_2_ of 30% saturation. α-Synuclein expression was induced with 0.2 mM isopropyl thiogalactopyranoside and the deuterated protein purified as described previously.^40^ The protein was used as a lyophilized powder.

### Amyloid fibril formation

α-Synuclein fibril formation was based on a previously described method.^34^ α-synuclein was dissolved in 20 mM sodium phosphate buffer HPCE pH 7.4 (Sigma Aldrich, UK) to give 500 μL aliquots at a final concentration of 500 μM. The solutions containing a micro polytetrafluoroethylene magnetic stirrer bar (Fisherbrand, UK) were incubated for 72 h at 40 °C with maximal stirring on a RCT Basic heat plate (IKA, Staufen, Germany).

### Amyloid seed generation

Amyloid fibril ‘seeds’ were generated using a liquid nitrogen freeze/thaw process repeated three times.^41^ The freeze/thaw process consisted of Eppendorf tubes containing 500 μL of amyloid fibrils being plunged into liquid nitrogen for 5 min before being removed and placed into a beaker of water to thaw.

### Transmission electron microscopy

TEM imaging was carried out using a JEOL JEM-2100 Plus (JEOL, Tokyo, Japan) operating at 200 kV. Samples were prepared using a previously reported method.^42^ Amyloid fibril seed solutions were first diluted to 0.01–0.5 mg ml^−1^ in Milli-Q water, before a small aliquot (5 μL) was pipetted onto a 300-mesh carbon-coated copper grid that had been glow discharged. The solution was left to adsorb for 30 s before a wedge of filter paper was used to wick the solution off the copper grid. The grid was then rinsed with distilled H_2_O after which an aliquot of 2% (w/v) uranyl acetate was applied and left to stain for 30 s. The stain was then wicked away using filter paper and the sample was left to air dry.

### Small angle neutron scattering, SANS2D, ISIS

SANS measurements were carried out at ambient temperature on the second-generation time-of-flight SANS2D instrument at ISIS (Harwell, UK). Sample solutions were measured in disc-shaped (“banjo”) quartz cells with a path length of 1 mm held in a rotating sample changer. An incident wavelength range of 2–14 Å with a sample-to-detector distance of 4 m, gave a *Q* range of *Q*_min_ = 0.002 Å^−1^ − *Q*_max_ = 3 Å^−1^. The SANS data collected was corrected for transmission, background, and pixel sensitivity of the 2-D detector, and averaged into a 1-D function, *I*(*Q*). Data was then scaled to absolute intensity for the scattering cross section per unit sample volume (cm^−1^) using Mantid.^43^ Data analysis and modelling was performed in SASView.^44^

#### Small angle neutron scattering kinetics

A solution containing hydrogenated α-synuclein seeds 1.2 mg ml^−1^ and deuterated α-synuclein monomer 2.5 mg ml^−1^ in 40% D_2_O (sodium phosphate HPCE buffer pH 7.4, 10 mM) was mixed, transferred to a rotating “banjo” cell (250 μL) and immediately measured at ambient temperature with an acquisition time of 4 h. Data were sliced into 30 min time slices using Mantid.^43^

#### Fitting small angle neutron scattering data

TEM images were used initially as a guide to particle shape for α-synuclein fibrils. A cylinder model utilizing as constraints dimensions determined from TEM images was used as the basis of the model applied to the SANS data. SASView was then used to optimize the model and improve the “goodness of fit”.^44^ The reduced chi-squared values from SASView were used as a measure of the “goodness of fit”; this parameter is related to the difference between the experimental data and the model. For a good fit to the data, the value of the reduced chi-squared will tend to 1.

## Author contributions

The manuscript was written through contributions of all authors. All authors have given approval to the final version of the manuscript.

## Conflicts of interest

There are no conflicts to declare.

## Supplementary Material

CB-002-D1CB00001B-s001
