# Supplementary material for: Elongation rate and average length of amyloid fibrils in solution using isotope-labelled small-angle neutron scattering
Source: RSC Chem Biol. 2021 Apr 14;2(4):1232–8. doi: 10.1039/d1cb00001b (PMC8341957; doi:10.1039/d1cb00001b)
Supplement: CB-002-D1CB00001B-s001 [file CB-002-D1CB00001B-s001.pdf]

Electronic Supplementary Material (ESI) for RSC Chemical Biology.
This journal is © The Royal Society of Chemistry 2021

# Electronic Supplementary Information

## Elongation rate and average length of amyloid fibrils in solution using isotope-labelled small-angle neutron scattering

Ben J. Eves,[†] James J. Doutch,[‡] Ann E. Terry,[§] Han Yin,[†] Martine Moulin,[⊥¥] Michael Haertlein[⊥¥], V. Trevor Forsyth,[⊥, ‖] Patrick Flagmeier, [#,¶] Tuomas P. J. Knowles, [#,¶] Gudrun Lotze,[§] Annela M. Seddon[∇] and Adam M. Squires[†*]

†Department of Chemistry, University of Bath, Bath, UK

‡ISIS Facility, Rutherford Appleton Laboratory, STFC, Chilton, Didcot OX11 0QX, UK

§MAX IV Laboratory, Lund University, P.O. Box 118, Lund 221 00, Sweden

⊥Life Sciences Group, Institut Laue Langevin, 38042 Grenoble Cedex 9, France

‖School of Chemistry & Physics, Keele University, Staffordshire ST5 5BG, UK

#Department of Chemistry, University of Cambridge, Cambridge, CB2 1EW, UK

¶Centre for Misfolding Diseases, University of Cambridge, Cambridge, CB2 1EW, UK

∇School of Physics, HH Wills Physics Laboratory, Tyndall Avenue, University of Bristol, Bristol BS8 1TL, UK

**Fibril Morphology**

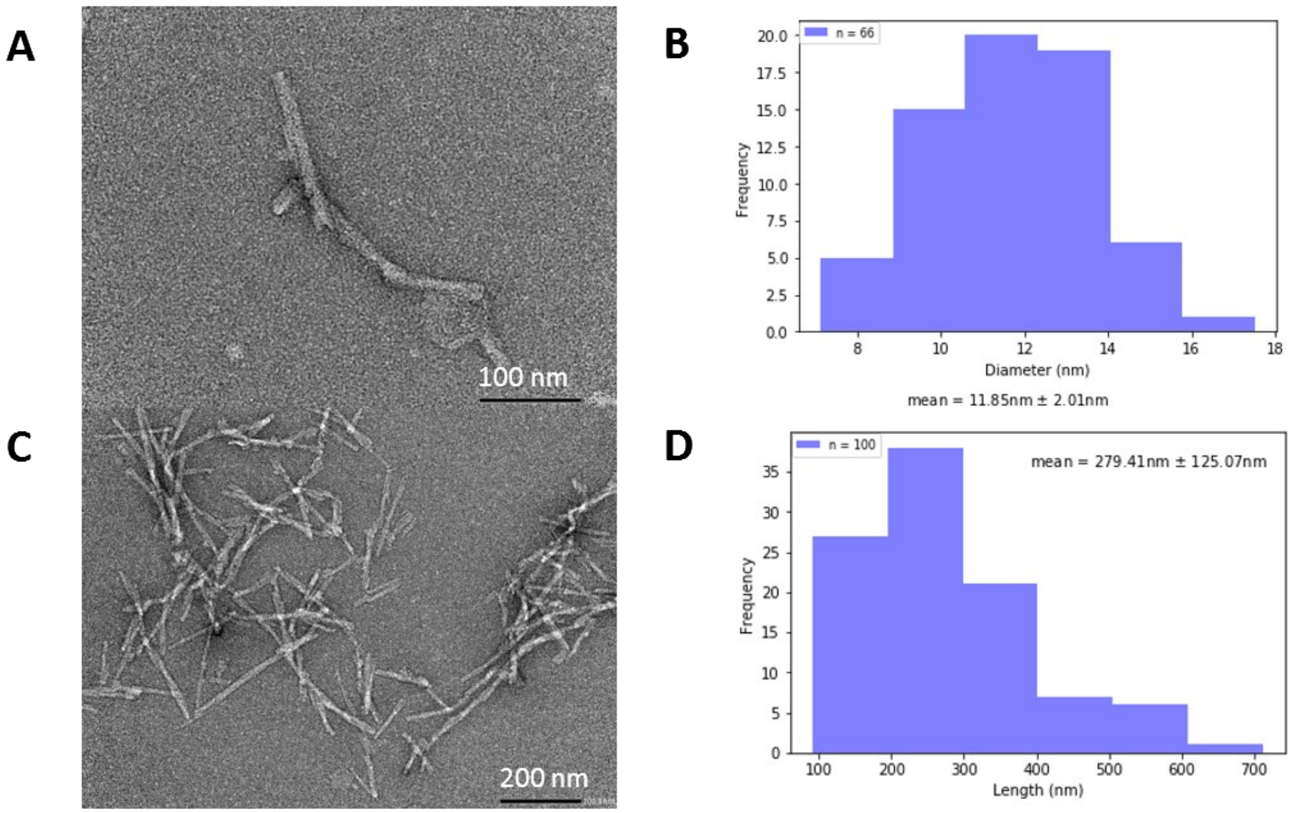


Figure S1. Fibril morphology of α-synuclein fibril seeds. (a) Zoomed view of α-synuclein seeds. (b) Histogram corresponding to the diameter of α-synuclein fibril seeds. Mean diameter was measured to be 12 ± 2 nm (n=100). (c) TEM image of α-synuclein fibril seeds showing varied seed length. (d) Histogram distribution of α-synuclein fibril seed length. Mean length was measured to be 279 ± 125 nm (n=66).

### Modified Guinier Plot

The forward scattering intensity of the cross-section $I_c(0)$ is given by a modified Guinier plot for elongated particles where *ln(Q I(Q))* is plotted versus $Q^2$,[36]

$$I(q) = \frac{I(0)}{q}\exp\left(-q^2\frac{R_g^{\,2}}{2}\right),$$

The y-intercept of the plot is $I_c(0)$. By determining the forward scattering intensity of the cross-section, a mass-per-unit length of a fibril,[45] *M* can be determined

$$M = \frac{1000 I_c(0) d^2 N_A}{\pi C(\rho_p - \rho_s)^2}$$

where *d* is the mean density of a protein (1.35 g $ml^{-1}$), $N_A$is Avogadro's number (6.023 × $10^{23}$ $mol^{-1}$), *C* is the concentration of peptide/protein in the fibril seeds, $\rho_p$is the scattering length density of the fibrils and , $\rho_s$ is the scattering length density of the solvent.

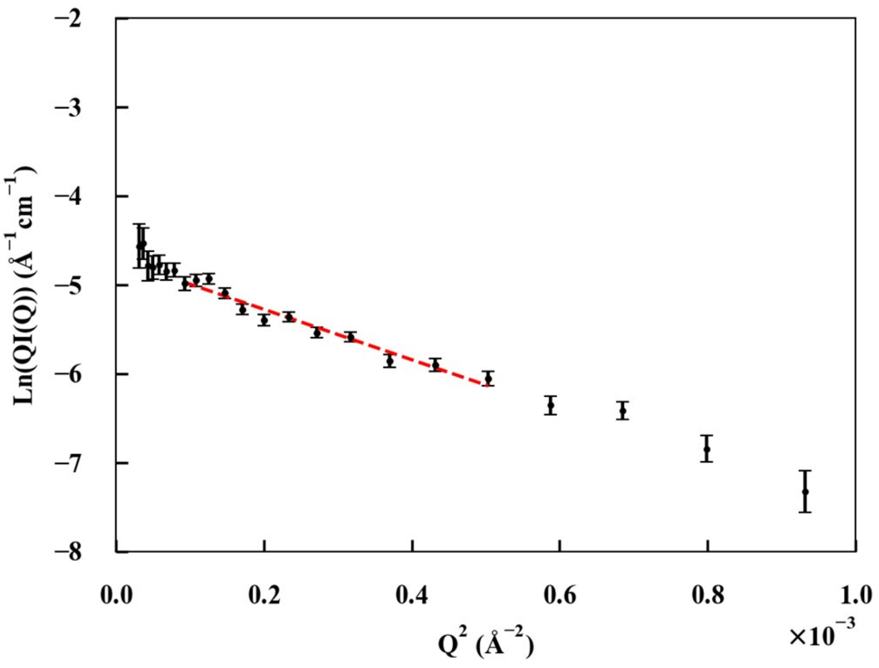


Figure S2. Modified Guinier plot of α-synuclein SANS profile.

### Contrast Match Series

To monitor only the growing deuterated ends of the fibrils, the scattering contribution from the hydrogenated seeds must be removed. When the scattering length density (SLD) of the solvent is matched to that of hydrogenated seeds, the contrast between the solvent and the seeds is zero. At this contrast match point the measured intensity from the seeds is zero. A contrast match series where the component to be matched is prepared at the same concentration in different mixtures of $H_2O/D_2O$ solvent can be used to determine the contrast match point.

The resultant scattering curves from hydrogenated α-synuclein seeds in 0, 20, 40, 60, and 80% $D_2O$ (2.5 mg $ml^{-1}$) are shown in Figure S3. Common practice in determining the contrast match point from a contrast match series is to plot the square root of the intensity at low *Q* versus the volume fraction of $D_2O$. Values obtained at negative contrast, where the SLD of the $H_2O/D_2O$ solvent is lower than the SLD of the α-synuclein seeds are treated as negative. Applying a linear regression to the resultant data gives rise to the volume fraction of $D_2O$ with minimal contrast (Figure S4). The contrast match point for hydrogenated α-synuclein seeds is $X_{D_2O}$ = 0.385. This value is in good agreement with the expected contrast match point for proteins, $X_{D_2O}$ = 0.4.[46]

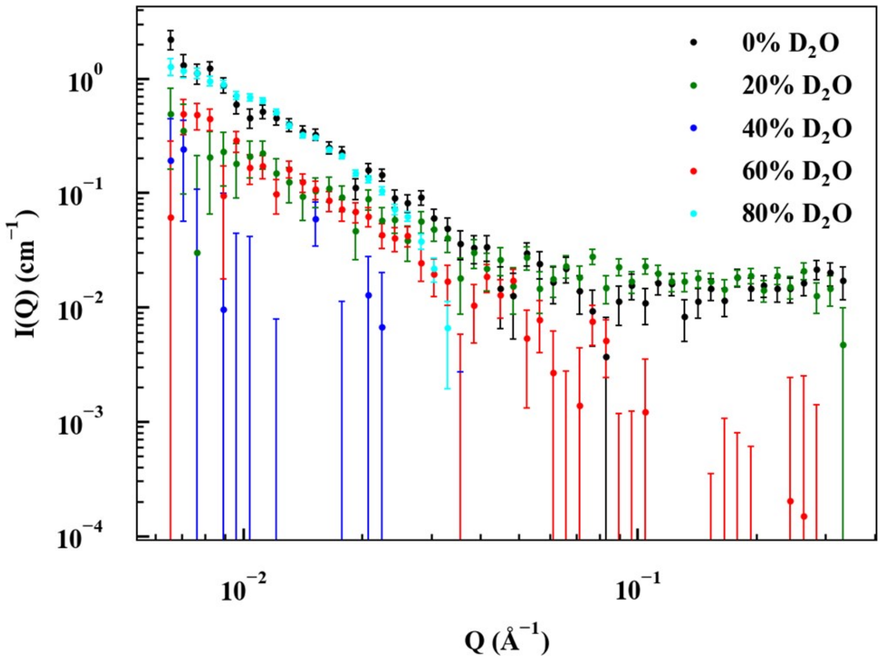


Figure S3: Contrast match series of hydrogenated α-synuclein fibrils (2.5 mg ml$^{-1}$) in varying $H_2O/D_2O$.

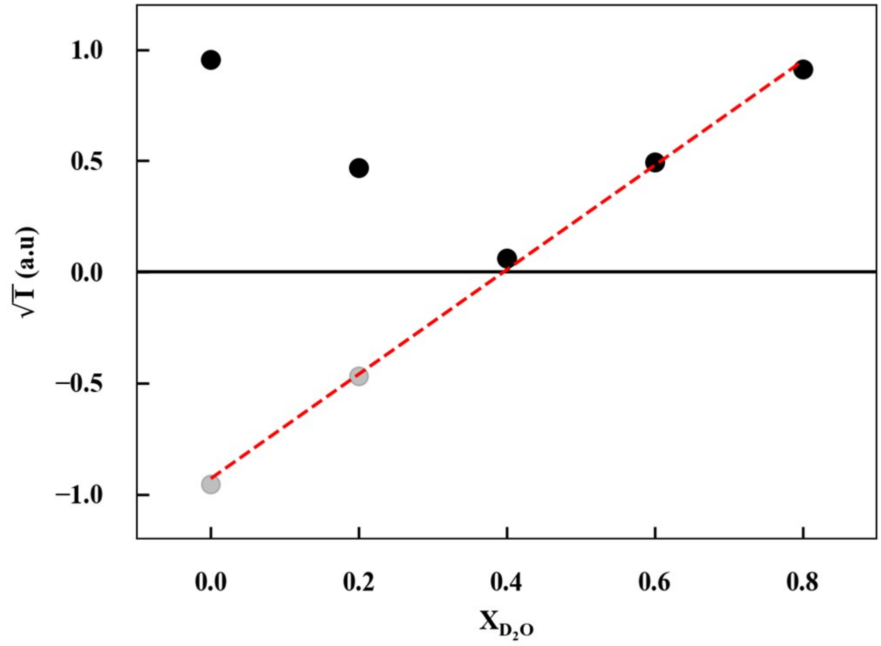


Figure S4: Square root of the intensity at low *Q* versus the volume fraction of $D_2O$ for hydrogenated α-synuclein fibrils. Values treated as negative (negative contrast) are shown in grey.

## References


(1) Guinier, A.; Fournet, G. *Small Angle Scattering of X-Rays*; Wiley: New York, 1955.

(2) Burkoth, T. S.; Benzinger, T. L. S.; Urban, V.; Morgan, D. M.; Gregory, D. M.; Thiyagarajan, P.; Botto, R. E.; Meredith, S. C.; Lynn, D. G. Structure of the β-Amyloid (10 - 35) Fibril. *J. Am. Chem. Soc.* **2000**, *122* (33), 7883–7889. https://doi.org/10.1021/ja000645z.

(3) Timmins, P.; Pebay-Peyroula, E.; Welte ', W. Detergent Organisation in Solutions and in Crystals of Membrane Proteins. *Biophys. Chem.* **1994**, *53*, 27–36.
